# Combined Experimental and DFT Study of Alumina (α-Al_2_O_3_(0001))-Supported Fe Atoms in the Limit of a Single Atom

**DOI:** 10.3390/nano15110804

**Published:** 2025-05-27

**Authors:** Ramazan T. Magkoev, Yong Men, Reza Behjatmanesh-Ardakani, Mohammadreza Elahifard, Ivan V. Silaev, Aleksandr P. Bliev, Nelli E. Pukhaeva, Anatolij M. Turiev, Vladislav B. Zaalishvili, Aleksandr A. Takaev, Tamerlan T. Magkoev, Ramazan A. Khekilaev, Oleg G. Ashkhotov

**Affiliations:** 1 Department of Physical Electronics, Herzen State Pedagogical University of Russia, 48 Moika Embankment, 191186 St. Petersburg, Russia; magkoev_r@mail.ru; 2 Laboratory of Physics of Adsorption Phenomena, Department of Condensed Matter Physics, North Ossetian State University, Vatutina 44-46, 362025 Vladikavkaz, Russia; bigjonick@yandex.ru (I.V.S.); bliev@mail.ru (A.P.B.); pukhaeva@jinr.ru (N.E.P.); ra6jt@mail.ru (A.M.T.); aleksandrtakaev@mail.ru (A.A.T.); rkhekilaev@mail.ru (R.A.K.); 3 School of Chemistry and Chemical Engineering, Shanghai University of Engineering Science, Shanghai 201620, China; 4 Department of Chemical Engineering, Faculty of Engineering, Ardakan University, Ardakan P.O. Box 184, Iran; behjatmanesh@pnu.ac.ir; 5 Division of Atmospheric Sciences, Desert Research Institute, Reno, NV 89512, USA; mohammadreza.elahifard@dri.edu; 6 Geophysical Institute—The Affiliate of Vladikavkaz Scientific Centre of the Russian Academy of Sciences, Markova 93a, 362002 Vladikavkaz, Russia; vzaal@mail.ru; 7 Institute of Informatics, Electronics and Robotics, Kabardino-Balkarian State University, Chernyshevskogo 173, 360004 Nal’chik, Russia; oandi@rambler.ru

**Keywords:** adsorption, thin films, oxide-supported metal particles, aluminum oxide, iron, surface-sensitive techniques

## Abstract

To probe the properties of single atoms is a challenging task, especially from the experimental standpoint, due to sensitivity limits. Nevertheless, it is sometimes possible to achieve this by making corresponding choices and adjustments to the experimental technique and sample under investigation. In the present case, the absolute value of the electronic charge the Fe atoms acquire when they are adsorbed on the surface of aluminum oxide α-Al_2_O_3_(0001) was measured by a set of surface-sensitive techniques: low-energy ion scattering (LEIS), Auger electron spectroscopy (AES), low-energy electron diffraction (LEED), and work function (WF) measurements, in combination with density functional theory (DFT) calculations. The main focus was the submonolayer coverage of Fe atoms in situ deposited on the well-ordered stoichiometric α-Al_2_O_3_(0001) 7 nm thick film formed on a Mo(110) crystal face. An analysis of the evolution of the Fe LVV Auger triplet upon variation of the Fe coverage shows that there is electronic charge transfer from Fe to alumina and that its value gradually decreases as the Fe coverage grows. The same trend is also predicted by the DFT results. Extrapolation of the experimental Fe charge value versus coverage plot yields an estimated value of a single Fe atom adsorbed on α-Al_2_O_3_(0001) of 0.98e (electron charge units), which is in reasonable agreement with the calculated value (+1.15e). The knowledge of this value and the possibility of its adjustment may be important points for the development and tuning of modern sub-nanometer-scale technologies of diverse applied relevance and can contribute to a more complete justification and selection of the corresponding theoretical models.

## 1. Introduction

Iron (Fe) as a nano-component of composite materials plays an essential role in a variety of applications, ranging from superconducting and magnetic material science and technology to heterogeneous catalysis [1,2,3,4,5,6,7,8,9,10,11,12,13,14,15,16,17,18,19,20,21,22,23,24,25,26]. Some such multicomponent materials are Fe atoms, nanoclusters, and thin films formed on the surface of metallic and metal oxide substrates [1,2,4,5,6,9,10,11,12,13,14,15,16,17,18,19,20,21,22,23,24,25,26]. The latter offers a vast number of possibilities to tune and control the properties of oxide-supported iron by adjusting the Fe cluster size, structure, and morphology; the oxide support atomic structure and stoichiometry; and the metal/oxide interface. In this sense, a quite detailed characterization of the Fe deposits on an Al_2_O_3_ surface, carried out by Arranz et al. [20], revealed the main trends determining the morphology, electronic state, and stability of an Fe/Al_2_O_3_ interface. It was found that at very low Fe coverage, there is notable charge transfer from the Fe atom to the oxygen of the substrate, diminishing as the Fe coverage grows. However, no evidence for Fe-Al orbital hybridization was found. Such charge transfers and redistribution determine the structure, morphology, and electronic properties of the Fe deposits and the metal/oxide interface. Similarly, Lehnert et al. [24] have shown that charge redistribution at the Fe/Al_2_O_3_ interface is a key point determining the growth mode of the Fe film. It has also been demonstrated that there is a threshold of Fe coverage, c.a. 0.2 monoatomic layers, on Al_2_O_3_ and MgO substrates, differentiating the ionic and atomic nature of adsorbed Fe species [26].

A quite strong interaction at the Fe/MgO(111) interface resulting in the oxidation of Fe was observed by Fonin et al. [23]. This is attributed to the pronounced effect of the electric field of the polar MgO(111) surface. The observed trend is in line with that observed in the earlier work of DiCastro et al. [14,25], demonstrated by synchrotron radiation-induced photoelectron spectroscopy; at the Fe/MnO interface at low Fe coverage, there is strong charge donation from Fe to the oxide support, weakening as the Fe coverage increases. Using the same technique, Nakajima et al. [19] have shown that on the surface of another easily reducible oxide, TiO_2_, low-coverage deposited Fe reduces Ti^4+^ to Ti^3+^ so that the resultant supported Fe clusters are of a non-metallic character. A similar reduction of Ce^4+^ to Ce^3+^ also occurs for ceria-supported ultrasmall Fe particles, yielding Fe_2_O_3_, with the effect enhancing soot combustion and CO oxidation over Fe/CeO_2_ [18]. Interface charge transfer and the phase and stoichiometry transformation of ZrO_2_ upon the deposition of Fe are also key points for ethane dehydrogenation reaction activity and selectivity [17]. The Fe deposited on NiO quite easily reduces the oxide to metallic Ni due to charge redistribution in the ternary Fe-Ni-O system at the meta/oxide interface [16]. The formation of a ternary Fe-Si-O system is also the point governing the Fischer–Tropsch reaction over an Fe/SiO_2_ catalyst, as has been recently discovered by Chang [13]. Contrary to the above observations, no charge transfer was detected in the Fe/MgO(100) system, as revealed by Fetzer et al. [12].

The general trend of the Fe deposits on the oxide surfaces is that there is charge transfer between the metal and the oxide, which is more pronounced in the case of reducible oxides (transition metal oxides) compared to non-reducible oxides (simple metal oxides). However, there are no reported data concerning the absolute value of the charge the Fe atoms acquire when chemisorbed at the metal oxide surface. At the same time, this value is considered to be a key point that determines the variety of physicochemical properties of the metal/oxide system. In relation to this, the aim of the present work was to determine the value of the charge of Fe atoms when they are deposited on the surface of α-Al_2_O_3_(0001). Aluminum oxide of this closely packed surface structure is chosen as a representative non-reducible and stable oxide with a presumably sharp metal/oxide interface with no metal/oxide surface mixing. From the experimental viewpoint, it is challenging to measure the charge of a single atom due to sensitivity limits. At the same time, it is this value that determines the specific properties of single Fe atoms of high applied relevance [3,8]. In the present study, by extending a recent short note [27], the obtained values of Fe charge are compared with the performed DFT studies. The novelty compared to a previous study [27] is as follows. Experimental data on the absolute values of charges of adsorbed atoms, and particularly single atoms, on the oxide surfaces, as well as the other substrates, are quite rare. In this regard, since there is almost no way to compare the obtained results with the results of other studies and assess their validity, it seems quite reasonable to strengthen the validity of these findings through additional experimental and theoretical investigations. One of the additional appropriate experimental tools used is a work function measurement technique that allows probing charge transfer/polarization at the interface. Combining this technique with theoretical studies enables a more detailed understanding and justification of the observed processes. It should be noted that the charge state is not the only parameter defining catalytic activity. Additionally, a variety of other parameters, such as local density of states at high spatial resolution, local atomic geometry and its dynamics, and interface states must be considered. These can be obtained through a combination of other sophisticated experimental and theoretical techniques. However, the focus of the present study is a specific case: the charge state of oxide-supported metal atoms. This parameter is highly relevant to various fundamental and applied aspects, especially in combination with the abovementioned and other parameters.

## 2. Experimental and Computational Details

The characterization techniques used are implemented in the VGS Escalab MkII system (VG Scientific, Birmingham, UK), enabling Auger electron spectroscopy (AES), low-energy ion scattering (LEIS), low-energy electron diffraction (LEED) and work function measurements in Anderson mode. For AES, a single-pass cylindrical mirror analyzer with coaxial electron gun, operating at a primary energy of 3 keV, was used (RBD Instruments, Bend, OR, USA). The spectra were recorded in dNE/dE mode, with the peak-to-peak amplitude taken as Auger intensity (I) [28]. For the acquisition of the LEIS spectra, the hemispherical retarding field analyzer (VG Scientific, Birmingham, UK) was used. He^+^ ions with energy of 1 keV were used as primary ions. The main feature of this technique is that it is inherently sensitive only to the content of the uppermost atomic layer of the substrate [28]. The surface atomic structure was monitored by the retarding rear-view hemispherical four-grid electron optics with a coaxial electron gun, operating at an energy range of 60 to 100 eV (Varian 981-2148, San Diego, CA, USA). The work function (ϕ) was measured by monitoring the contact potential difference with the aid of a low-energy electron gun (Physical Electronics PHI, York, UK) operating at primary energy not exceeding 10 eV, using the approach known as Anderson mode of the work function measurement [28]. Since this mode is sensitive only to the relative variation of the work function, the work function of bare Mo(110), chosen as a substrate, was considered as a reference—5.0 eV [29].

The aluminum oxide film was formed in situ on the Mo(110) surface by the well-known procedure of reactive evaporation of metallic aluminum in an oxygen ambient at a partial pressure of normally not exceeding 10^−6^ mbar [30]. In this case, the alumina film was formed by thermal evaporation of aluminum from the Knudsen cell at an evaporation rate of about 1.25 × 10^13^ cm^−2^s^−1^, while maintaining the Mo(110) substrate temperature at 700 K and an oxygen partial pressure at 5 × 10^−7^ mbar. After the film thickness reached approximately 7 nm, the evaporation of Al was stopped, but the oxygen exposure at the given pressure continued for 5 min. As a result, well-ordered α-Al_2_O_3_(0001) film is formed due to the well-matched lattice parameter with Mo(110) [30]. The aluminum evaporation flux rate was measured by a quartz microbalance mounted coaxially to the Mo(100) on the sample holder. Thus, given the amount of Al atoms being deposited on the Mo(110), the alumina film thickness is estimated by the approach proposed by Goodman [30]. The Fe was deposited in situ on the substrate by thermal evaporation from a separate Knudsen cell. Using the quartz microbalance, the evaporation flux was controllably maintained in the range of (1.7–2.5) × 10^12^ cm^−2^s^−1^. Taking into account the surface concentration of atoms on the Mo(110) surface (1.47 × 10^15^ cm^−2^) and the ratio of atomic radii of Mo and Fe (0.145 nm and 0.140 nm, respectively), the close-packed Fe film on Mo(110) corresponds to a surface concentration of 1.53 × 10^15^ cm^−2^. The latter was considered as corresponding to a coverage (θ) of one monolayer (ML) (θ = 1 ML).

All materials used (the metals and the oxygen gas) are of high research grade quality to ensure clean experimental conditions. Prior to evaporation, the Al and Fe metal sources were carefully outgassed in the UHV chamber, ensuring that no impurities were detected by AES when these metals were deposited on the Mo(110) substrate. To obtain an atomically clean Mo(110) sample, it was initially annealed at 1300 K in an oxygen ambient (partial pressure: 10^−6^ mbar) to remove possible carbon and sulfur contamination, followed by hydrogen exposure to remove oxygen from the sample. After that, prior to the formation of alumina film, the Mo(110) underwent a series of high-temperature “flashings” at 2600 K to ensure maximal atomic cleanliness of the substrate. Experimental techniques and procedures are described in more detail elsewhere [27,31,32].

For the calculations, the ASE-SIESTA package was used to optimize the structures via periodic density functional theory [33,34]. The force and SCF convergence thresholds were defined to be less than 0.05 eV/Å and 10^−5^ eV, respectively. The BFGS algorithm was used for force minimization [35]. Unrestricted spin was considered for solving the Schrödinger equation during all optimization and post-processing calculations. A double zeta plus polarization (DZP) basis set for valence electrons, norm-conserving pseudopotentials for core electrons, and the revised PBE (*rPBE*) functional were chosen for all ASE-SIESTA calculations [36,37]. The average binding energy of Fe atoms on the aluminum oxide surface is defined as follows:

(1) $$E_{bind}^{avg}=\left(E_{{Al}_{2}O_{3}/nFe}-nE_{Fe}-E_{{Al}_{2}O_{3}}\right)/n$$

where *n* is the number of Fe atoms, $E_{Fe}$ is the total energy of a single Fe atom in the gas phase, $E_{{Al}_{2}O_{3}}$ is the total energy of the pristine surface of Al_2_O_3_, and $E_{{Al}_{2}O_{3}/nFe}$ is the total energy of the *n* Fe atoms deposited on the Al_2_O_3_ surface. The atomic charges were calculated using the Bader code [38,39] with cube files generated by the full potential FHI-aims program [40,41,42,43]. K-grids of 6 × 6 × 2 and 4 × 4 × 1 for bulk and slab models, respectively, were generated using the Monkhorst–Pack algorithm [44] to sample the first Brillouin zone. Scalar relativistic effects within the zero-order regular approximation were considered in all FHI-aims calculations. It is noteworthy that among the used algorithms (Mulliken, Hirshfeld, and Bader’s atoms-in-molecules), the latter provides the best agreement between experiment and theory. The Bader analysis is based purely on the electron density, where the density is partitioned according to its zero-flux surfaces. By defining the adatom/substrate interface plane as a zero-flux surface and comparing it to the experimentally measured charge (derived from Auger transition probability, which is proportional to the charge density of the respective shell), a direct correlation between experimental and theoretical values can be established.

## 3. Results and Discussion

**Figure 1:**
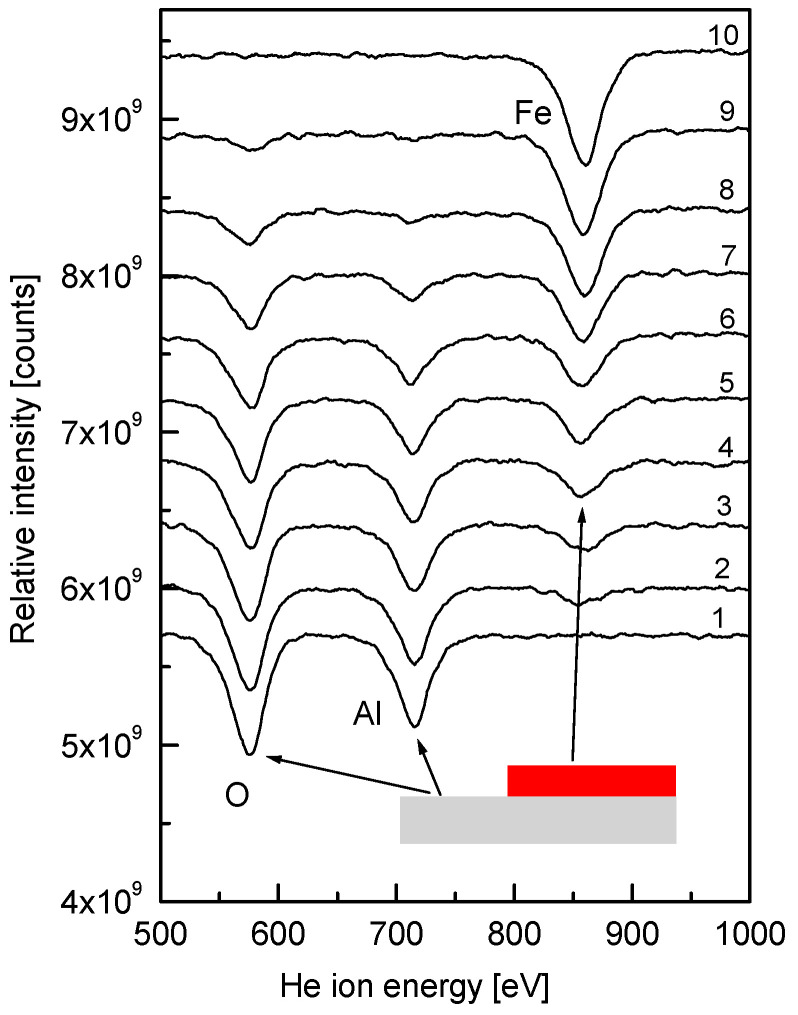
Low-energy ion scattering spectra for Fe coverage growth on α-Al_2_O_3_(0001). Fe coverage, ML: 1—0; 2—0.1; 3—0.2; 4—0.3; 5—0.4; 6—0.5; 7—0.6; 8—0.8; 9—0.9; 10—1. Substrate temperature: 300 K.

**Figure 2:**
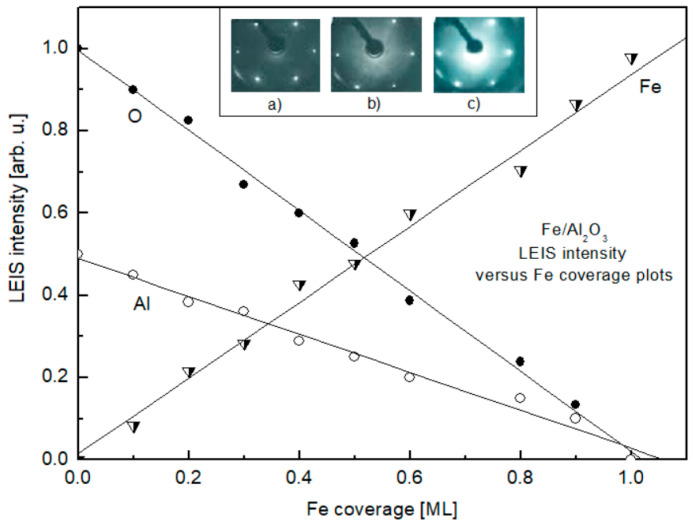
Oxygen, aluminum and iron LEIS intensities versus Fe coverage plots for Fe on α-Al_2_O_3_(0001). Inlay: Low-energy electron diffraction patterns for bare Mo(110) (**a**), α-Al_2_O_3_(0001)/Mo(110) (**b**), Fe/α-Al_2_O_3_(0001)/Mo(110) (**c**). Alumina film thickness is 7 nm, Fe coverage is 1 ML. Substrate temperature: 300 K.

The key issue when considering the formation of an Fe overlayer on α-Al_2_O_3_(0001) is to determine the growth mode of the metal. To distinguish between the 2D- or 3D-mode of formation of the Fe film in the submonolayer region (i.e., when θ < 1 ML), direct information can be obtained by LEIS due to its inherent sensitivity to the content of only the uppermost layer of the sample [27,28]. The transformation of the LEIS spectra upon the stepwise deposition of Fe on α-Al_2_O_3_, held at room temperature, in the submonolayer coverage range has been presented in our recent study [27] and is replotted in a different form here and discussed in more detail for the context of the present study (Figure 1). Prior to Fe deposition, the spectrum consists of two lines at He^+^ scattering energies of 576 eV and 715 eV, corresponding to oxygen and aluminum, respectively (spectrum 1). Beginning from the lowest investigated coverage of 0.1 ML, an additional line at 860 eV, corresponding to Fe, emerges (spectrum 2). Subsequent increase in Fe coverage results in the gradual decrease in the intensity of lines, corresponding to Al and O, with a concomitant increase in the intensity of the Fe line (spectra 3–10). Spectrum 10 consists of only one line, corresponding to Fe, with no detectable Al and O features. Taking into account the specificity of the LEIS technique, this is clear evidence that spectrum 10 corresponds to the Fe film completely covering the substrate with only underlying alumina exposed. Measuring the intensities of the lines as the distance between the maximum and the baseline of the corresponding line and plotting them versus the Fe coverage yields the dependences shown in Figure 2. The observed linear plots indicate that the Fe film grows in a 2D-mode, at least until the one monolayer coverage is achieved (θ = 1 ML). To monitor the possible atomic superstructure of the Fe film on the α-Al_2_O_3_(0001) the LEED measurements were performed. Initially, the LEED pattern of the bare Mo(110) surface was detected, and is shown in the inlay of Figure 2a. It consists of hexagonally arranged spots corresponding to Mo(110) symmetry. The same symmetry is seen for the 7 nm thick α-Al_2_O_3_(0001) film formed on Mo(110) (Figure 2b) with the position of diffraction spots very close to those of Mo(110). This is the result of the close lattice parameter match between the α-Al_2_O_3_(0001) and Mo(110) surfaces [30]. Deposition of Fe atoms on the α-Al_2_O_3_(0001) results in the increase in the LEED background intensity with no extra diffraction spots in the entire submonolayer region studied (Figure 2c). This means that the Fe atoms deposited on the surface of alumina do not acquire long-range structural order. The latter is in contrast with the Fe growth on the metal substrates, where a number of different superstructures are observed, beginning from the very submonolayer region and up to multilayers [15]. It is reasonable to attribute this difference to the nature of bonding of Fe atoms to the metal and the metal oxide surface, which differentiates metal–substrate, lateral metal–metal interaction, surface migration, and the parameters determining atomic structuring.

**Figure 3:**
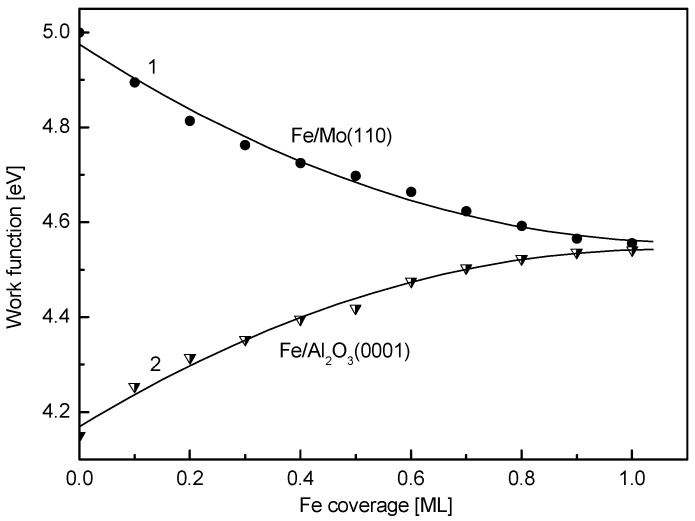
The work function versus coverage plots upon stepwise deposition of Fe on the Mo(110) (curve 1) and the α-Al_2_O_3_(0001) surface (curve 2).

This difference is also demonstrated by notably different work function versus coverage plots in the submonolayer region for bare Mo(110) and α-Al_2_O_3_(0001) substrates, shown in Figure 3. When Fe is deposited on Mo(110), the work function gradually decreases from 5.0 eV to 4.55 eV at a coverage of approximately 1 ML (curve 1). The decrease in the electron work function upon adsorption of atoms on the surface of the substrate is usually attributed to the formation of a positive dipole in the adatom/substrate complex, with the positive part being directed outside the substrate surface (polarization of chemisorption bond from adatom to substrate) [45]. Contrary to Mo(110), growth of Fe coverage on α-Al_2_O_3_(0001) results in an increase in the work function (Figure 3, curve 2). Beginning from the value of 4.15 eV, characteristic for bare alumina, the work function versus coverage plot gradually increases until the same stationary value as for Mo(110)—4.55 eV, is also achieved at θ ≈ 1 ML. The latter corroborates the above LEIS results, indicating the formation of a complete 2D monolayer of Fe on α-Al_2_O_3_(0001). Otherwise, for a 3D growth mode, the work function value would have been stabilized at a notably higher Fe coverage, when the 3D islands coalescence to completely cover the substrate surface. According to the above dipolar model of the work function change, the increase in the value of ϕ when Fe is deposited on the alumina surface may be attributed to the chemisorption charge polarization from the substrate to Fe, meaning the Fe atoms on the surface of α-Al_2_O_3_(0001) acquire a negative charge. This, however, contradicts the subsequent AES results, indicating the opposite trend.

**Figure 4:**
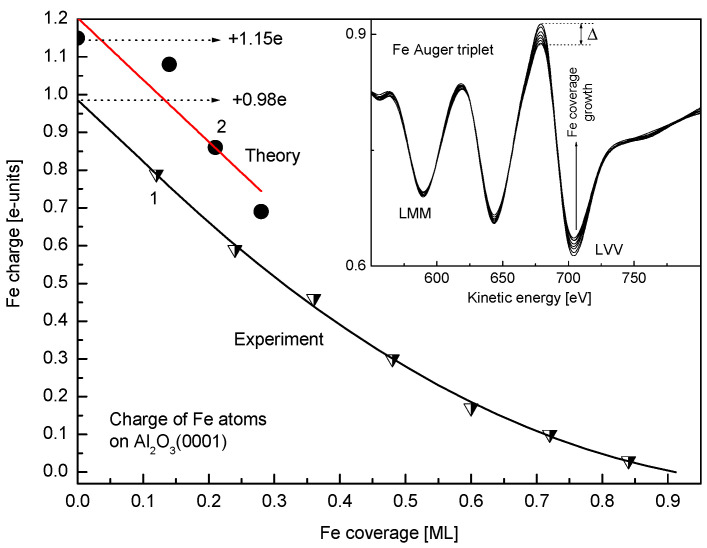
(Inlay) The baseline normalized Fe LMM Auger spectra for Fe on α-Al_2_O_3_(0001) for Fe coverage stepwise growth from 0.12 ML to 0.96 ML. Curves 1 and 2 are experimental and theoretical values of the charge of Fe atoms versus coverage, respectively. Extrapolation of curve 1 to zero coverage gives an estimated value of the charge of single Fe adatom.

The series of baseline normalized Auger spectra for Fe on α-Al_2_O_3_(0001) in the Fe LMM-triplet region, recorded during stepwise Fe coverage growth in the range between 0.12 ML and 0.96 ML, are shown in the inlay of Figure 4. The main focus of the present study was to monitor not the details of the spectra but the relative variation of intensities of the components of this triplet—LVV (LM_45_M_45_) (702 eV) and LMM (LM_23_M_23_) (589 eV). Thus, according to the well-established approach [28], the spectra were recorded with a high time constant of the spectrometer and were normalized to the baseline to enable more reliable comparison and clearly observe the disproportional growth of LVV and LMM intensities with the increasing Fe coverage. This was carried out in order to assess the evolution of the Fe valence charge upon adsorption of Fe atoms on α-Al_2_O_3_(0001), based on the fact that the Auger probability is proportional to the charge value of the level involved in the Auger transition. For the 3D-metals, the following relationship between the ratio (R) of LM_45_M_45_ and LM_23_M_23_ intensities (I) and the valence charge (q) holds [46]:

R = I(LM_45_M_45_)/I(LM_23_M_23_) = Const q (q − 1) (2)

when the value of the valence charge changes by Δq, this relationship acquires the following form:

R = Const (q + Δq) (q + Δq − 1)(3)

The latter allows one to determine the excess charge the Fe atoms acquire upon adsorption on α-Al_2_O_3_(0001). The corresponding plot of the charge value of the Fe adatom versus Fe coverage is shown in Figure 4 (curve 1). It is seen that, unlike the trend expected from the work function versus coverage plot (Figure 3, curve 2), the Fe atoms acquire positive charge, i.e., the chemisorption bond is polarized towards the alumina substrate. The inconsistency between the AES and the work function results may be attributed to the fact that, unlike in the metallic substrate, when the dipolar model prevails, for band gap materials, like alumina, the work function is an interplay between the band bending and Fermi level shift upon metal adsorption [45,47]. As seen in Figure 4 (curve 1), the charge of Fe adatoms gradually decreases with the increasing coverage. The solid line, as the best fit to the experimental points, approximated to zero coverage (θ → 0), gives an estimated value of the charge of single Fe atom adsorbed on the surface of α-Al_2_O_3_(0001), which is +0.98e.

**Figure 5:**
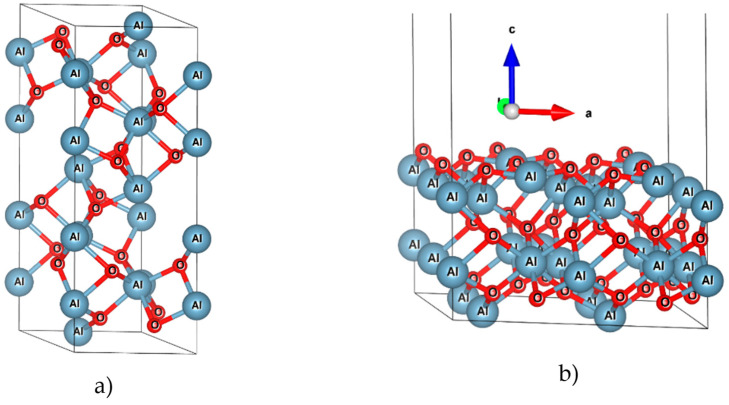
Bulk phase of α-Al_2_O_3_ (**a**) and the slab model of Al_2_O_3_ (0001) surface (**b**). Data show that the surface layer atoms undergo reconstruction.

To theoretically assess this value, first, the optimized structure of the bulk phase of α-Al_2_O_3_(0001) and the slab model of the Al_2_O_3_(0001) surface were built (Figure 5). The optimized lattice constants of the bulk structure are *a* = *b* = 4.842 Å and *c* = 13.177 Å, which are comparable to the experimental values of *a* = *b* = 4.754 Å and *c =* 12.990 Å [48] with less than a 2% error. Each oxygen atom in the bulk structure of Al_2_O_3_ is bonded to four Al atoms. All four bond lengths are not equal; two bond lengths (2.005 Å) are longer than the other two bond lengths (1.88 Å). These bond lengths are slightly longer than those in the experimental structure, which has values of 1.99 Å and 1.87 Å, respectively. The optimization of the slab model shows that the outermost surface layer reconstructs so that the oxygen atoms form only three bonds with different bond lengths to the Al inner layer. The bond lengths are 1.73 Å, 1.81 Å, and 1.90 Å, which are smaller than the bond lengths in the bulk structure.

**Figure 6:**
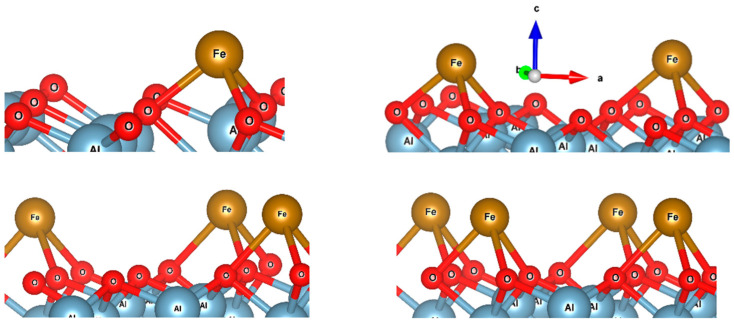
The optimized structures of one, two, three, and four Fe atoms deposited on Al_2_O_3_ (0001) surface. Fe atoms prefer hollow sites and connect to three adjacent oxygen atoms.

With the main focus on calculating the charge of a single Fe atom deposited on the Al_2_O_3_(001) surface, the calculations are limited to only a few simple cases: four, three, two, and finally one Fe atom on the surface. Figure 6 shows the optimized structures of these cases. The data show that each Fe atom prefers to occupy the hollow site and interacts with three oxygen atoms. The Fe-O bond length increases as the concentration of Fe atoms on the Al_2_O_3_(0001) surface increases. For the single Fe atom deposited on the Al_2_O_3_ surface, the average Fe-O bond length is equal to 1.96 Å, while the average Fe-O bond length for the four-Fe case is equal to 2.19 Å. Therefore, it can be concluded that the interaction of Fe with oxygen atoms is stronger for a single Fe deposited on Al_2_O_3_(0001) than for the four-Fe case, so the charge of Fe decreases from the single Fe to the four-Fe case. Table 1 shows the calculated average QTAIM charge and binding energy of Fe atoms on the surface of Al_2_O_3_(0001) for different numbers of atoms, corresponding to the indicated mean coverage. The latter is estimated as the ratio of the number of adsorbed Fe atoms (1 to 4) to the number of adsorption sites (14) for the built slab model. As is clear, Fe atoms are electron donors to the acceptor oxygen atoms. With an increasing number of Fe atoms, their average electron donation decreases. Binding energy data show that for a single Fe on the Al_2_O_3_ surface, the interaction is strongest. These data are in line with the above experimental data predicting that the charge of Fe atoms is positive and increases with decreasing Fe concentration. This trend is quantitatively evident in the similar experimental and calculated Fe charge versus coverage plots (Figure 4, curves 1 and 2). With a single atom limit, the experimental and calculated values of charge (+0.98e and +1.15e, respectively) are in reasonably good agreement with each other. To rationalize the charge transfer mechanism, one can consider above results; even though alumina is not an easily reducible oxide, there is a reconstruction of the α-Al_2_O_3_(0001) surface, so that, unlike the bulk, where oxygen is bound to four Al atoms, the oxygen binds to three Al atoms on the surface, thus opening an additional possibility for Fe to bind to the outermost “released” oxygen atom. Further, as Fe, according to the above DFT results, prefers to bind to a hollow site and interact with not one but three oxygen atoms, the overall Fe → O charge transfer is enhanced. The seeming contradiction between the results presented in Figure 3 and Figure 4 can be explained as follows. According to the widely accepted Helmholtz model, the observed increase in the work function upon Fe adsorption on alumina (Figure 3) is due to the formation of a negative surface dipole moment, assuming that the electron charge transfers from alumina to Fe. However, Figure 4 and DFT results show the opposite trend. In order to reconcile these seemingly contradictory results, it should be taken into account that, in contrast to the simpler case of the Fe-Mo(110) system, the resulting dipole moment of the Fe-Al2O3 surface layer is created by a superposition of the dipole moments of the Fe-O interfacial bond and the Al-O bond of alumina substrate. As shown above, due to symmetry breaking, the Al-O distance at the surface is shortened, and the O atom binds to three Al atoms instead of four Al atoms in the bulk. The adsorbed Fe atom, to a certain extent, restores the local symmetry, characteristic of bulk alumina, substituting one of the missing Al atoms. This leads to an increase in the Al-O interatomic distance to the values close to that of bulk alumina, simultaneously increasing the charge of the O atoms due to donation from Fe. These two effects result in an increase in the negative dipole moment of the outermost layer of the alumina, which may exceed the value due to the opposite change in the dipole moment caused by Fe. Thus, averaging the opposite effects caused by the formation of a positive dipole moment of adsorbed iron atoms, on the one hand, and an increase in the magnitude of the negative dipole moment due to an increase in the bond length and the magnitude of the O charge, on the other hand, leads to a resulting increase in the measured value of the work function. Moreover, adsorption of atoms may cause a shift of the Fermi level in the band gap of alumina, thus changing its location relative to the vacuum level, monitored by the Anderson method as a contact potential difference variation.

In fact, knowledge of the absolute value of the atomic charge of a metal atom on the oxide surface contributes to a more complete understanding of the characteristics of the corresponding metal/oxide system, and the possibility of varying this with coverage allows controlled adjustment of the system parameters for certain practical applications. In addition, the possibility of comparing this value with the results of calculations can contribute to a more complete justification and selection of the corresponding theoretical models.

## 4. Conclusions

In an attempt to estimate the absolute electronic charge acquired by a single metal atom adsorbed on the oxide surface, a case study of Fe on α-Al_2_O_3_(0001) was conducted by means of combined experimental and DFT studies. The Fe/Al_2_O_3_(0001) model system has been prepared in a controlled manner in ultra-high vacuum and characterized in situ by a set of surface science techniques. including Auger electron spectroscopy, low-energy ion scattering, low-energy electron diffraction, and work function measurements. The substrate consistent of a well-ordered stoichiometric α-Al_2_O_3_(0001) thin film (7nm thick) grown on a Mo(110) crystal plane by reactive evaporation of Al in an oxygen ambient. The main focus was to probe the growth mode and the electronic state of Fe on alumina at submonolayer coverage. At a growth rate of 0.05 monolayers per minute and room temperature, a 2D growth mode was observed for Fe. Analysis of the Fe LVV Auger triplet during the growth of Fe film indicates an electronic charge transfer from Fe to alumina. The extent of the transferred charge gradually decreases as the Fe coverage increases. Extrapolation of the Fe charge versus coverage plot to zero coverage yields the value of electronic charge +0.98 e for a single Fe atom adsorbed on the α-Al_2_O_3_(0001) surface, which is in reasonable agreement with the DFT results (+1.15e). Moreover, both experiment and theory qualitatively agree that the charge of Fe atoms on alumina decreases with increasing surface concentration. Knowledge of the absolute charge value of an oxide-supported single atom is important for emerging ‘single-atom’ technologies. Moreover, the ability to adjust this value, for instance, by varying the coverage, offers an opportunity for fine tuning of their relevance for specific applications.

## Figures and Tables

**Table 1:** The average charge and binding energy of *n* × Fe atoms on Al_2_O_3_(0001) surface. All energy values are in eV.

*n*	Mean Coverage	Charge	Binding Energy
1	0	+1.15	−6.76
2	0.14	+1.08	−5.12
3	0.21	+0.86	−4.47
4	0.28	+0.69	−3.89

## Data Availability

The original contributions presented in this study are included in the article. Further inquiries can be directed to the corresponding authors.

## References

[1] Ravensburg A.l., Grassi M.P., Hjörvarsson B., Kapaklis V. (2024). Effect of iron layer thickness on the interlayer exchange coupling in Fe/MgO (001) superlattices. Phys. Rev. B.

[2] Reyes A., Herrera G., Capiod P., Le Roy D., Dupuis V., Cañero-Infante I., Saint-Girons G., Bachelet R., Resta A., Ohresser P. (2024). Preferential orientations of FeRh nanomagnets deposited on a BaTiO_3_ epitaxial thin film. Phys. Rev. B.

[3] Berwanger J., Polesya S., Mankovsky S., Ebert H., Giessibl F.J. (2020). Atomically resolved chemical reactivity of small Fe clusters. Phys. Rev. Lett..

[4] Beyazit Y., Beckord J., Zhou P., Meyburg J.P., Kühne F., Diesing D., Ligges M., Bovensiepen U. (2020). Local and nonlocal electron dynamics of Au/Fe/MgO(001) heterostructures analyzed by time-resolved two-photon photoemission spectroscopy. Phys. Rev. Lett..

[5] Costentin C., Drouet S., Robert M., Savéant J.-M. (2012). A local proton source enhances CO_2_ electroreduction to CO by a molecular Fe catalyst. Science.

[6] Colonna S., Ronci F., Cricenti A., Luches P., Valeri S., Qi J., Xu Y., Miller J.K., Tolk N. (2007). Ferromagnetic–antiferromagnetic Fe/NiO(100) interface studied by non-linear Kerr effect. Surf. Sci..

[7] Luque R., Lin C.S.K., Wilson K., Clark J. (2016). Handbook of Biofuels: Production Processes and Technologies.

[8] Zhang P., Chen H.-C., Zhu H., Chen K., Li T., Zhao Y., Li J., Hu R., Huang S., Zhu W. (2024). Inter-site structural heterogeneity induction of single atom Fe catalysts for robust oxygen reduction. Nat. Commun..

[9] Egawa C., Onawa K., Iwai H., Oki S. (2004). Interaction of CO and NO with Fe thin films grown on Rh(100) surface. Surf. Sci..

[10] Li X., Luo Y., Wu S., Lian H., Deng X. (2023). The exceptional performance of the plasmonic Au-Fe/TiO_2_ nanocatalysts achieved by O plasma activation. Catal. Today.

[11] Luches P., Torelli P., Benedetti S., Ferramola E., Gotter R., Valeri S. (2007). Structure and electronic properties of Fe nanostructures on MgO(001). Surf. Sci..

[12] Fetzer C., Dezsi I., Szucs I., Tancziko F., Balogh A.G. (2009). The interaction of Fe on MgO(100) surfaces. Surf. Sci..

[13] Chang Q., Li J., Suo H., Qing M., Wang H., Zhang C., Wen X., Xiang H., Yang Y., Li Y. (2024). Unravelling the formation of Fe_2_SiO_4_ on Fischer-Tropsch Fe/SiO_2_ catalyst. Catal. Today.

[14] Di Castro V., Ciampi S. (1995). XPS study of the growth and reactivity of Fe/MnO thin films. Surf. Sci..

[15] Kołaczkiewicz J., Bauer E. (2000). V and Fe on the W(110) face. Surf. Sci..

[16] Masi R., Reinicke D., Müller F., Steiner P., Hüfner S. (2002). The interface of Mn, Fe, Co and Au metal films on NiO(001), investigated by photoemission and low energy electron diffraction. Surf. Sci..

[17] Zheng Y., Zhang X., Li J., An J., Zhu X., Li X. (2023). Role of active oxygen species in Fe-doped ZrO_2_ catalyst during CO_2_ assisted ethane dehydrogenation reaction. J. Catal..

[18] Rodriguez M., Leonardi S.A., Hanon F., Miró E.E., Milt V.G., Gaigneaux E.M. (2024). Plasma-assisted deposition of Mn and Fe phases on CeO_2_ biomorphic fibers for soot combustion and CO oxidation. Catal. Today.

[19] Nakajima N., Kato H., Okazaki T., Sakisaka Y. (2004). Photoemission study of the modification of the electronic structure of transition-metal overlayers on TiO_2_ surfaces: I. Fe onTiO_2_(110). Surf. Sci..

[20] Arranz A., Perez-Dieste V., Palacio C. (2002). Growth, electronic properties and thermal stability of the Fe/Al_2_O_3_ interface. Surf. Sci..

[21] Li S., Wang F., Xie Z., Ng D., Shen B. (2023). A novel core-shell structured Fe@CeO_2_-ZIF-8 catalyst for the reduction of NO by CO. J. Catal..

[22] Luches P., Benedetti S., Liberati M., Boscherini F., Pronin I.I., Valeri S. (2005). Absence of oxide formation at theFe/MgO(001) interface. Surf. Sci..

[23] Fonin M., Dedkov Y.S., Rüdiger U., Güntherodt G. (2007). Growth and morphology of the epitaxial Fe(110)/MgO(111)/Fe(110) trilayers. Surf. Sci..

[24] Lehnert A., Krupski A., Degen S., Franke K., Decker R., Rusponi S., Kralj M., Becker C., Brune H., Wandelt K. (2006). Nucleation of ordered Fe islands on Al_2_O_3_/Ni_3_Al(111). Surf. Sci..

[25] Di Castro V., Polzonetti G., Ciampi S., Contini G., Sakho O. (1993). Photoemission study of the Fe/MnO interface formation. Surf. Sci..

[26] Magkoev T.T., Vladimirov G.G., Remar D., Moutinho A.M.C. (2002). Comparative study of metal adsorption on the metal and the oxide surfaces. Solid State Commun..

[27] Magkoev T.T., Men Y., Behjatmanesh-Ardakani R., Takaev A.A., Khekilaev R.A. (2025). The value of charge of Fe atoms adsorbed on the surface of a-Al2O3(0001). Pis’ma Zhurnal Tekhnicheskoi Fiz..

[28] Yates J.T. (2015). Experimental Innovations in Surface Science: A Guide to Practical Laboratory Methods and Instruments.

[29] Berge S., Gartland P.O., Slagsvold B.J. (1974). Photoelectric work function of a molybdenum single crystal for the (100), (110), (111), (112), (114), and (332) faces. Surf. Sci..

[30] Goodman D.W. (1996). Chemical and spectroscopic studies of metal oxide surfaces. J. Vac. Sci. Technol. A.

[31] Magkoev T.T., Men Y., Behjatmanesh-Ardakani R., Elahifard M., Abaev V.T., Chalikidi P.N., Zaalishvili V.B., Magkoev T.T., Ashkhotov O.G. (2024). The value of charge of Fe single to multiple atoms doped in Ge: Combined experimental and density functional theory study. Solid State Commun..

[32] Magkoev T.T. (2021). Effect of electron tunneling through the oxide film grown on metal substrate upon the efficiency of molecular reaction over the oxide supported metal nanoparticles: A case of CO oxidation on Au/Al_2_O_3_/Mo(110). Vacuum.

[33] García A., Papior N., Akhtar A., Artacho E., Blum V., Bosoni E., Brandimarte P., Brandbyge M., Cerdá J.I., Corsetti F. (2020). Siesta: Recent developments and applications. J. Chem. Phys..

[34] Larsen A.H., Mortensen J.J., Blomqvist J., Castelli I.E., Christensen R., Dułak M., Friis J., Groves M.N., Hammer B., Hargus C. (2017). The atomic simulation environment-a Python library for working with atoms. J. Phys. Condens. Matter.

[35] Fischer T.H., Almlof J. (1992). General methods for geometry and wave function optimization. J. Phys. Chem..

[36] Hammer B., Hansen L.B., Nørskov J.K. (1999). Improved adsorption energetics within density-functional theory using revised Perdew-Burke-Ernzerhof functionals. Phys. Rev. B.

[37] Van Setten M.J., Giantomassi M., Bousquet E., Verstraete M.J., Hamann D.R., Gonze X., Rignanese G.M. (2018). The PseudoDojo: Training and grading a 85 element optimized norm-conserving pseudopotential table. Comp. Phys. Commun..

[38] Yu M., Trinkle D.R. (2011). Accurate and efficient algorithm for Bader charge integration. J. Chem. Phys..

[39] Henkelman G., Arnaldsson A., Jónsson H. (2006). A fast and robust algorithm for Bader decomposition of charge density. Comp. Mater. Sci..

[40] Blum V., Gehrke R., Hanke F., Havu P., Havu V., Ren X., Reuter K., Scheffler M. (2009). Ab initio molecular simulations with numeric atom-centered orbitals. Comp. Phys. Commun..

[41] Havu V., Blum V., Havu P., Scheffler M. (2009). Efficient O(N) integration for all-electron electronic structure calculation using numeric basis functions. J. Comp. Phys..

[42] Marek A., Blum V., Johanni R., Havu V., Lang B., Auckenthaler T., Heinecke A., Bungartz H.-J., Lederer H. (2014). The ELPA library: Scalable parallel eigenvalue solutions for electronic structure theory and computational science. J. Phys. Condens. Matter.

[43] Yu V.W., Corsetti F., García A., Huhn W.P., Jacquelin M., Jia W., Lange B., Lin L., Lu J., Mi W. (2018). ELSI: A unified software interface for Kohn–Sham electronic structure solvers. Comp. Phys. Commun..

[44] Monkhorst H.J., Pack J.D. (1976). Special points for Brillouin-zone integrations. Phys. Rev. B.

[45] Luo Y., Tang Y., Chung T.F., Tai C.-L., Chen C.-Y., Yang J.-R., Li D.Y. (2021). Electron work function: An indicative parameter towards a novel material design methodology. Sci. Rep..

[46] Allen G.C., Tucker P.M., Wild R.K. (1977). High resolution LMM auger electron spectra of some first-row transition elements. Surf. Sci..

[47] Esser N., Benne I., Srama R., Richter W. (1992). The surface dipole contribution to the work function for Sb on GaAs(110): A comparative study by Kelvin probe and Raman spectroscopy. Surf. Sci..

[48] Ishizawa N., Miyata T., Minato I., Marumo F., Iwai S. (1980). A structural investigation of [alpha]-Al_2_O_3_ at 2170 K. Acta Cryst. B.

